# Tactile Discrimination, Praxis and Cognitive Impulsivity in ADHD Children: A Cross-Sectional Study

**DOI:** 10.3390/ijerph17061897

**Published:** 2020-03-14

**Authors:** Dulce Romero-Ayuso, Donald Maciver, Janet Richmond, Sara Jorquera-Cabrera, Luis Garra-Palud, Carmen Zabala-Baños, Abel Toledano-González, José-Matías Triviño-Juárez

**Affiliations:** 1 Department of Physical Therapy, Faculty of Health Sciences, University of Granada, Occupational Therapy Division, Avda de la Ilustración n 60, 18016 Granada, Spain; 2 School of Health Sciences, Queen Margaret University Edinburgh, Edinburgh, Scotland EH21 6UU, UK; DMaciver@qmu.ac.uk; 3 School of Medical and Health Sciences, Edith Cowan University, 270 Joondalup Drive, Joondalup, WA 6027, Australia; inscribeot@outlook.com.au; 4 Centro de Terapia Infantil-AYTONA, C/Virgen de las Viñas n 16, 28031 Madrid, Spain; sara@aytona.com; 5 Faculty of Health Sciences, University of Castilla-La Mancha, Avda de la Real Fábrica de la Seda s/n., 45600 Talavera de la Reina, Spain; Luis.Garra@uclm.es (L.G.-P.); Carmen.Zabala@uclm.es (C.Z.-B.); Abel.Toledano@uclm.es (A.T.-G.); 6 Sistema Andaluz de Salud, Centro de Salud Zaidín Sur, C/ Poeta Gracián n 7, 18007 Granada, Spain; jmtjuarez@hotmail.com

**Keywords:** attention deficit hyperactivity disorder, tactile discrimination, cognitive impulsivity, praxis

## Abstract

Background: The study of attention deficit hyperactivity disorder (ADHD) has traditionally focused on deficit of inhibitory control and cognitive impulsivity. However, the pathophysiology of ADHD has also been associated with the somatosensory cortex. The aim of this study was to explore if there were differences in tactile discrimination and praxis between neurotypical and ADHD children and whether these differences could be explained by cognitive impulsivity. Methods: A cross-sectional study was conducted. The sample comprised 74 children aged 7 to 11 years divided in two groups: 43 with neurotypical development, 31 with ADHD. To assess tactile discrimination, the finger localization and the graphestesia tests were used. Praxis was assessed with the Kaufman Assesment Battery for Children (K-ABC) hand movement subtest, the action program and the Zoo Map subtests of the Behavioral Assessment of Dysexecutive Syndrome, and the complex figure of Rey–Osterrieth test (ROCF). Cognitive impulsivity was assessed using the Magallanes Computerized Impulsivity Scale test (EMIC). Results: Children with ADHD showed greater cognitive impulsivity (*p* = 0.038) and scored lower in Zoo Map (*p* = 0.023) and hand-movement subtests (*p* = 0.002), and in ROCF test (*p* = 0.004). Differences in praxis skills still remained after controlling by gender and cognitive impulsivity. Conclusion: Praxis deficit might have repercussions not only on the characterization of ADHD but also on its treatment.

## 1. Introduction

Attention deficit hyperactivity disorder (ADHD) is characterized by three main symptoms: inattention, hyperactivity, and impulsivity [1]. Most studies about ADHD have focused on determining whether ADHD is an executive function disorder and concluded that the key deficit of ADHD is the inhibitory control and cognitive impulsivity [2,3,4,5,6,7]. Recent research has focused on the role of motor circuits and sensory processing on the pathophysiology of ADHD [8,9,10,11,12,13], showing that the somatosensory cortex is thinner in children with ADHD. This might be related to the fact of high comorbidity with motor coordination disorders [14,15] that exceeds 50% of children with ADHD [15]. Luria and Piaget indicated that executive functions depend on more basic skills, such as sensorimotor, which develop at an early age [16,17]. More recently, other authors have pointed out that motor skills are not isolated skills and may be affected by other cognitive skills, especially with executive functions [18,19,20,21]. However, there is a lack of studies that try to examine whether the deficit of inhibitory control and cognitive impulsivity could be related to worse tactile perception and motor clumsiness in ADHD children.

In this sense, cognitive impulsivity can be understood as a predisposition to react quickly to internal or external stimuli without taking into account the negative consequences of these reactions, thus associating with a lack of self-control, linked to neuropsychological mechanisms that imply a dysfunctional inhibition of thoughts and behaviors [22,23]. The measurement of response latency (reaction time) is crucial to understanding cognitive impulsivity [24]. Several authors [25,26,27] consider that the cognitive impulsivity is a key factor in the executive functions of a subject and children with greater cognitive impulsivity show, among other aspects, a worse inhibition of responses. Deficit in the inhibition of responses is manifested by impulsive behaviors, such as responding before the task is understood, responding before having all the information available, losing attention by irrelevant stimuli, or making inappropriate responses [28,29].

In relation to tactile perception, Parush et al. [30] and Mailloux et al. [31] found that children with ADHD had poorer performance in tasks of tactile discrimination. Tactile discrimination contributes to the development of an accurate and precise body schema, which is necessary for planning new actions [32,33]. Proprioception and tactile discrimination are important for appropriate development of the body schema, which is the basis for the development of praxis [34]. Proprioception could be understood as the ability to recognize the position of each part of the body at any given moment, thus allowing the reception/integration of information, interpreting and executing a response to the sensory stimuli received [35]. Praxis is defined as the mental process through which a certain action that involves movement is carried out, being intrinsically connected with sensory feedback in the development of the action [35]. In this sense, praxis is a process oriented towards an objective and is determined by different mechanisms [36]: ideation (creativity and motivation to carry out an action plan), planning (organization of the sequence of movements), sequencing (order of actions), and execution (motor ability to finish the action) [37]. Therefore, it is understood that praxis has two major components, a motor component and a cognitive one, which although they work together to achieve a common goal, are independent processes and have been associated with different brain regions [38]. Studies about sensory processing have shown that difficulties in tactile discrimination impact on the ability to identify similarities and differences between stimuli, especially with regards to their temporal and spatial characteristics [39]. Sensory processing implies the ability for the detection, modulation, interpretation, and internal organization of sensory information to execute appropriate responses to the demands of the context [15,40]. Skin mechanoreceptors, which are pressure-sensitive, are involved in tactile discrimination [41,42]. Tactile information facilitates perception of the spatial properties of objects, such as their size and shape, and is used to plan how the hand grips an object during handling, in association with visual information about the object [43,44]. Praxis and tactile discrimination are essential for the performance of activities of daily living and learning (writing and manipulative activities), including the ability to learn, organize and maintain an appropriate level of activity [45]. Sensorimotor processing discrepancies may be partially responsible for motor learning and control difficulties [15]. The detection of praxis and tactile discrimination deficit is essential, since it can affect the daily and emotional life of children with ADHD, who are considered a vulnerable group owing to their lack of social skills, low self-esteem and poor sense of competitiveness [45,46,47,48].

Research shows that children with ADHD have deficits in sensory modulation, evaluated primarily through proxies, through questionnaires to parents or teachers about their responses to sensory stimuli [49]. However, the literature that tries to verify whether these deficits reported by caregivers are due to a deficit in discrimination and praxis is scarce. Taking into account previous studies as well as research on the human connectome [50,51], the question considered is whether the deficit in inhibitory control or cognitive impulsivity could be related to a more impulsive and less planned response in children with ADHD, which could affect the execution of the praxis.

In the light of the above, the aim of this study was to explore if there were differences in tactile discrimination and praxis between children with neurotypical development and children with ADHD and whether these differences could be explained by cognitive impulsivity.

## 2. Materials and Methods

### 2.1. Design

A cross-sectional study was performed.

### 2.2. Participants and Procedure

Participants were selected using nonprobabilistic convenience sampling and they were divided into two groups for comparison: a group of children whose development was neurotypical and a group of children with ADHD [50]. The inclusion criteria for the children in the neurotypical group were as follows: age between 7 and 11 years; no history of neurological or psychological disorder; no behavioral or cognitive problems; nonfulfillment of the DSM-IV-TR criteria for ADHD; no psychopharmacological, psychological, and/or psychoeducational treatment; and an intelligence quotient (IQ) ≥ 80 [51]. The inclusion criteria for children in the ADHD group were as follows: age between 7 and 11 years; an IQ ≥ 80; fulfillment of the DSM-IV-TR criteria for ADHD; and absence of other neurological illness. In addition, children with ADHD could not be receiving pharmacological treatment or must have discontinued treatment at least 24 h prior to assessment. All children receiving medical treatment were taking methylphenidate. The 7 to 11 year age range was chosen, as it is the period in which the diagnosis is first made [52].

The children in the neurotypical group were recruited from a public Infant and Primary School in Talavera de la Reina, Spain. We first contacted with the head teacher and the parents’ association to explain the project. After this, a letter was sent to the parents of children aged 7–11 years. The number of children initially included in the neurotypical group was 46, but three were excluded because of suspicion of ADHD after the administration of EDAH (scale for the assessment of ADHD children, validated for the Spanish population) [53]. In these cases, it was recommended they were assessed at the Child and Adolescent Mental Health Unit of Nuestra Señora del Prado Public Hospital (Talavera de la Reina, Spain). Regarding the ADHD group, children aged 7–11 years were recruited from the ADHD Association in Talavera de la Reina, Spain, and their parents were informed about the project by a psychologist. The number of children in this group was initially 33, but one was excluded owing to suspected autism spectrum disorder, and another was found to have an oppositional defiant disorder. Finally, 74 children were included in the study, 43 in the neurotypical group and 31 in the ADHD group. All participants were recruited between January and February 2017 and were assessed between February and May 2017. Once the project was completed, a personalized report was made for each child and the main researcher met with the parents to explain the results.

### 2.3. Instrumentation

In order to determine the intelligence quotient (IQ) and to know if the children met the inclusion criteria in relation to an IQ ≥ 80, four subtests (arithmetic, vocabulary, block design, and object assembly) were used from the revised version of the Wechsler Intelligence Scale for Children, which is the gold standard for assessment of intelligence [54]. It can be administered to children aged 6 to 16 years. The IQ score obtained using these subtests correlates highly with the score obtained using the complete scale (*r* = 0.93–0.95) [55,56,57]. Assessment time is thus reduced (a major advantage when several tests have to be administered). The mean score for the general population is 100 (*SD* = 15); the higher score the higher intelligence. To assess handedness, it was administered the short version of the Edinburgh Handedness Inventory (EHI) [58]. It has been widely used in children and adults [59,60,61].

#### 2.3.1. Cognitive Impulsivity

In order to assess cognitive impulsivity, it was used the EMIC task (Magellan Scale of Computerized Impulsivity), which is an adaptation of the task Matching Familiar Figure Test (MFFT), with the difference that it is administered with a computer and uses 16 new items [24]. For each item, the child has to look at the top of the computer screen, where a model is showed. Separated by a horizontal line, six figures are presented in the screen, five of them similar to the model and another one identical to this one. The child has to identify the identical figure to the model presented previously, placing the cursor on it. The program registers for each element the total number of errors and the response latency in milliseconds. If the answer is right, a smiling yellow face appears in the center of the screen, but if it has been wrong, a sad white face appears, and up to five more attempts are allowed. Before starting the test, four examples are made to check if the child has understood the instructions and knows how to handle the mouse. The shorter the response latency time, the greater the cognitive impulsivity.

#### 2.3.2. Tactile Discrimination

To assess tactile discrimination, two tests were used: Finger Localization Test [62,63] and Graphesthesia Test from the Luria-Nebraska Neuropsychological Battery—Children’s Revision [64]. In the Finger Localization Test, the examiner lightly touches a finger between the first and second joint while the child’s eyes are closed. The thumb is number one and the little finger is number five. The child must indicate by number or finger which finger was touched. The child then has to identify pairs of fingers touched simultaneously without visual input. We used a modified version of 20 items, which score ranges from 0 to 20 [65]; a higher score indicates a better finger localization. This test can be applied in children aged 6–13 years. In the administration of the Luria-Nebraska Graphesthesia Test, the child’s eyes are closed, the examiner draws a shape (cross, triangle, or circle) with a pencil on the back of the child’s hand, and the child is required to recognize it. The score ranges from 0 to 16; the higher score, the better tactile discrimination.

#### 2.3.3. Praxis

Regarding praxis, each of the four processes that comprise it (ideation; planning; sequencing; and execution) were assessed as follows:

##### Ideation

In order to assess ideation, it was administered the Action Program Subtest from the Behavioral Assessment of the Dysexecutive Syndrome (BADS) [66,67]. The Action Program Test is applied to determine action planning and problem solving skills [67]. In this test, the child is given a transparent cylindrical shape jar fixed with a removable transparent cover with a small hole in the center. There is also a test tube with a small piece of cork at the bottom. On the left of this tube, there is a metal bar (roughly L-shaped), which is not long enough to reach the cork, and a small container with a screw cap on its side and the top placed unscrewed and next to it. The child is prompted to pull the cork out of the tube using any of the objects in front of him/her, although he/she must lift them without touching them with the fingers. The score ranges from 0 to 5; a higher score, a better planning ability.

##### Planning

Planning was assessed using Zoo Map (ZM) subtest from the Behavioral Assessment of the Dysexecutive Syndrome (BADS). The Zoo Map subtest is a paper and pencil task that requires the child to plan a route in order to visit different zoo locations. The use of colored pens provides visual guidance to aid a child in recognizing where he/she has been, and a new route is required. We took into account the number of correct visits in the zoo, with a range score from 0 to 7. A higher score corresponds to better planning skills. Related to BADS, there is a version available since 2003 and it can be applied in children aged 8–16 years [68]. However, we used the adult version because, according to its normative data tables, it can be applied to children aged 7 and older (our sample comprised children aged 7 to 11 years). These norms are available in the November 2006 handbook (p. 39; normative tables) [67].

##### Sequencing

Regarding sequencing, it was assessed using a subtest of the *Kaufman Assessment Battery for Children* (K-ABC) [69] called Hand Movements (HM), which can be applied in children aged 2–12 years; in Hand Movements subtest, the administrator makes a series of taps on the table with his/her fist, palm, or side of the hand and the child copies the series of taps. The score ranges from 0 to 21, and a higher score indicates better praxis.

##### Execution

In order to assess execution, it was administered the Rey–Osterrieth complex figure test (ROCF) [70]. In the ROCF [70], the child is shown a drawing that he/she has to copy, with no time limit. It allows assessing organization, planning, and motor execution and strategies for solving problems as well as visual-constructional capacity. The score ranges from 0 to 36. The higher score, the better praxis skills.

All of these tests were administered by the same qualified neuropsychologist, who was trained in their administration. The assessment was conducted individually in a specially allocated room, in two separated sessions, each lasting 45 min. The first session included the EHI, Wechsler Intelligence Scale for Children—Revised (WISC-R) subtest, and Hand Movements subtest (K-ABC) that were administered following this sequence. The second session included the administration of EMIC, the Graphesthesia test, ROCF, Zoo Map-A (BADS), and Finger Localization and Action Program (BADS). If the child showed difficulties in finishing the sessions or maintaining attention, the assessment was interrupted and he/she was invited to rest for 10–15 min.

### 2.4. Ethics

The study was approved by the Ethics Committee of Nuestra Señora Del Prado Hospital (code 1/2017) and was carried out following the rules of the Declaration of Helsinki and the current data protection law. Parents of children of both groups gave their written informed consent.

### 2.5. Data Analysis

Data were examined for normality using visual inspection and Kolmogorov–Smirnov test (with the Lilliefors correction). Descriptive statistics were used to describe qualitative and quantitative variables (mean and standard deviation or median and Interquartile Range (IR), if they followed a normal distribution or not, respectively). Fisher’s exact test was performed to analyze differences in gender and hand dominance between the two groups of comparison (neurotypical group and ADHD group). To analyze differences in quantitative variables with normal and non normal distribution, the Student *t* test for independent samples or the Mann–Whitney U test were used, respectively.

It was conducted an analysis to know if there were differences between neurotypical children and ADHD children in age, gender, hand dominance, IQ, and cognitive impulsivity. To answer the aim of the study, it was initially performed a bivariate analysis to know if there were differences in Finger Identification and Graphesthesias tests (which are measures of Tactile Discrimination) and in ZM, Action Program, ROCF, and HM (which are measures of Praxis) between groups. Because the gender and cognitive impulsivity were distributed differently between groups, linear regression models were conducted to study the impact of the interaction of these variables with those tests of Tactile Discrimination and Praxis with differences between groups in the bivariate analysis. As these interactions were found to be nonsignificant (*p* > 0.05), analysis of covariance (ANCOVA) was subsequently performed with those tests of Tactile Discrimination and Praxis in which significant differences were found, introducing gender and cognitive impulsivity as confounders. The level of statistical significance was set at *p* ≤ 0.05. The statistical analysis was conducted using SPSS version 26 (IBM Corp., Armonk, NY, USA).

## 3. Results

### 3.1. Characteristics of the Sample

The median age of the children was 8.11 (IR 7.88–9) years. A total of 62.20% (*n* = 46) were male. Right hand dominance was 86.50% (*n* = 64). The mean of IQ was 104.08 (*SD* = 13.30). By group, there was a significantly higher proportion of male in ADHD group than in neurotypical group (*p* = 0.029). With respect to cognitive impulsivity, the reaction time was shorter in ADHD children than in neurotypical (*p* = 0.038). No statistically significant differences were found in age, hand dominance, and IQ. Table 1 shows the values of previous variables in both groups.

### 3.2. Differences in Tactile Discrimination and Praxis between Groups

Related to tactile discrimination, no statistically significant differences were found between groups in Finger identification (*p* = 0.133) and Graphestesias (*p* = 0.099) tests (Table 2). Regarding Praxis, the ADHD group scored lower than the Neurotypical group in planning, sequencing, and execution, being these differences statistically significant (*p* = 0.023, *p* = 0.002 and *p* = 0.004, respectively). No statistically significant differences were found between groups in ideation (*p* = 0.719) (Table 2).

Interactions of gender and cognitive impulsivity with planning, sequencing and execution were non statistically significant. Therefore, gender and cognitive impulsivity were subsequently included as confounders in each multivariate model performed. Thus, the final multivariate models showed that the ADHD group scored lower in planning (−2.13, *p* = 0.014), sequencing (−2.34, *p* = 0.002), and execution (−5.44, *p* = 0.004) than neurotypical group (Table 3).

## 4. Discussion

The current study aim was to explore whether children with ADHD showed deficits in tactile discrimination and praxis (given that there is a high comorbidity like coordination problems and motor clumsiness in these children) and whether these differences could be explained by cognitive impulsivity, thus could improve our understanding of ADHD. The results found that ADHD children had a lower performance compared with children with neurotypical development in praxis tests, specifically in planning (ZM), sequencing (HM), and execution (ROCF) of a purposeful activity, differences that still remained after controlling by gender and cognitive impulsivity. However, no statistically significant differences were found in the process of ideation of the praxis and in tactile discrimination (measured with Finger Identification and Graphesthesia tests). Likewise, in the bivariate analysis, differences were found between groups in cognitive impulsivity, although these differences disappeared after introducing this variable in the multivariate analysis as a confounder.

Most studies on ADHD focus on clarifying whether this disorder is an executive function disorder and on identifying which executive function is deficient [2,3,4,5,6,7]. However, research on the relationship between tactile perception, praxis, inhibitory control, and impulsivity in ADHD children is scarce [10]. However, there is a high comorbidity between ADHD and coordination problems and motor clumsiness in childhood. Therefore, it is relevant to view ADHD not purely as a deficit in executive functioning; it might be that more fundamental problems such a sensory and motor deficits underlie ADHD. Most of the studies that have tried to study sensory processing in children with ADHD have done it indirectly, through proxies, questionnaires to caregivers or teachers. From the results of these studies it has been concluded that children with ADHD have difficulties in sensory modulation.

The results of the present study do not allow us to conclude that children with ADHD have deficits in tactile discrimination, for the recognition of their fingers and graphesthesias. These results contrast with those found in a study of Parush et al. [30], in which it was observed that children with ADHD showed difficulties in identifying their fingers. It is possible that we could not find these differences because of the sample size of our study. Given that the results in graphesthesia show marginally significant differences, it might be interesting to conduct the study with a larger sample.

It has been found in praxis that children with ADHD performed worse in planning (ZM), sequencing (HM), and execution (ROCF). It has been postulated that motor anomalies in children with ADHD are manifestations of abnormal neural activity, especially in the primary motor cortex and superior parietal cortex, thus suggesting that children with ADHD use the primary motor cortex to perform simple motor tasks less than neurotypical children. These findings coincide with those of neuroimaging studies of children with ADHD [71], which could suggest impaired capacity to utilize the posterior parietal systems necessary for forming images to orient them in the correct motor sequence of finger movement.

On the one hand, the results could suggest a new explanation related to the prevalence of motor problems in school children with ADHD, which can be 30%–50% [14]. Also, the findings could explain a less fine motor coordination in these children [72]. On the other hand, it has been described that worse motor skills during the first 5–6 years of life might predict the subsequent onset of ADHD symptomatology, and the combination of worse motor skills and motor coordination disorders have been related to a poor evolution of ADHD [73,74]. Furthermore, a longitudinal study has found that up to 58% of subjects with ADHD and motor coordination disorders showed poor evolution and the presence of both disorders was the most important predictor of psychological malfunction in adolescence [75].

Regarding the relation between cognitive impulsivity and praxis, the findings suggested that cognitive impulsivity seems not to explain the differences in praxis between ADHD children and children with neurotypical development. In general, children with dyspraxia understand the goal for actions but are often incapable of planning their actions effectively [76]. Their movements frequently exhibit poor timing, are not scaled to the task, and/or are inappropriate to the demands of the task [76]. Lack of pre-movement readiness, anticipation, and organization affect their movement behavior adversely, so that their responses appear delayed or excessive [76]. Taking into account this, we consider our results of interest because the assessment and treatment of praxis deficit could improve the performance and participation of ADHD in their daily life.

Our research has a number of limitations, thus the findings should be interpreted with caution. First, the limitation of the study itself, since it is an observational design (which implies a greater risk of confounding variables that have not been taken into account) and a cross-sectional study (which does not demonstrate causal relations). Second, it is possible there might have been a bias for selection. Participants were recruited using nonprobabilistic convenience sampling, so that it could be difficult to generalize the results of this type of procedure to a larger population, although this sampling technique is useful in exploratory studies such as ours [77]. Third, the generalization of our results to a larger population could be limited by the sample size. Consequently, future research should be based on larger samples. Fourth, the coefficients of determination (adjusted R square) of the multivariate models were small, which may be due, among others, to the small sample size and the reduced number of independent variables introduced in the model [78]. Therefore, it would be appropriate to design new studies with larger sample sizes with other variables not assessed in this study. Fifth, the administrators were not blind to the children’s diagnosis. Sixth, we did not include a complete assessment of tactile perception. Thus, future studies should also include assessment of haptic perception, pressure, vibration, and pain, as well as an analysis of the proprioceptive and vestibular system to determine whether there is a relationship between tactile deficits and difficulties in proprioception.

## 5. Conclusions

Children with ADHD showed a lower performance in praxis, which could relate to a greater difficulty in learning and conducting tasks requiring fine motor skills, such as writing and other manual activities. These findings seem not to be related to cognitive impulsivity. We consider our results of interest because the detection of these motor problems can help us to design a specific treatment that could improve the prognosis of ADHD.

## Figures and Tables

**Table 1:** Sociodemographic characteristics, intelligence quotient, hand dominance, and cognitive impulsivity by group.

Characteristics	Neurotypical	ADHD	*p* Value
	Median (IR)	Median (IR)	
Age (years)	8.11 (8–9)	8.30 (7.10–9.60)	0.852 ^1^
Cognitive impulsivity	17.85 (13.11–24.33)	13.80 (7.80–21.43)	0.038 ^1^
	Mean (*SD*)	Mean (*SD*)	
IQ	104.93 (12.71)	102.09 (14.19)	0.521 ^2^
	*n* (%)	*n* (%)	*p* Value
Gender			
Female	21 (48.80%)	7 (22.60%)	0.029 ^3^
Male	22 (51.20%)	24 (77.40%)	
Hand dominance			
Left	4 (9.30%)	6 (19.40%)	0.304 ^3^
Right	39 (90.70%)	25 (80.60%)	

ADHD: Attention Deficit Hyperactivity Disorder; IR: Interquartile Range; IQ: Intelligence Quotient; ^1^ Mann–Whitney U test; ^2^ Student *t* test; ^3^ Fisher’s exact test.

**Table 2:** Differences between groups in tactile discrimination and praxis.

Characteristics	Neurotypical	ADHD	*p* Value
	Median (IR)	Median (IR)	
Tactile discrimination			
Finger Identification	19 (18–20)	18 (17–20)	0.133 ^1^
Graphestesias	14 (13–16)	14 (12–15)	0.099^1^
Praxis			
Ideation (Action Program)	3 (0–4)	3 (0–4)	0.719 ^1^
Planning (ZM)	2 (1–8)	1 (−1–3)	0.023 ^1^
Sequencing (HM)	15 (12–17)	12 (10–14)	0.002 ^1^
Execution (ROCF)	32 (29.75–35)	30 (22.75–33)	0.004 ^1^

ADHD: Attention Deficit Hyperactivity Disorder; IR: Interquartile Range; ZM: Zoo Map A—Behavioral Assessment Disexecutive Syndrome; HM: Hand Movements—K-ABC; ROCF: Rey–Osterrieth Complex Figure Test; ^1^ Mann–Whitney U test.

**Table 3:** Results of multivariate analysis of planning, sequencing, and execution.

**Planning (ZM)**	**β**	**Standard Error**	***p*-Value**	**95% Confidence Interval**	
				**Lower Limit**	**Upper Limit**
Intercept	1.43	1.40	0.313	−1.37	4.23
Group (ADHD)	−2.13	0.85	0.014	−3.82	−0.44
Gender (female)	−0.57	0.85	0.502	−2.27	1.12
Cognitive impulsivity	0.03	0.04	0.474	−0.05	0.11
R2 adjusted: 0.07					
**Sequencing (HM)**	**β**	**Standard Error**	***p*-Value**	**95% Confidence Interval**	
				**Lower limit**	**Upper limit**
Intercept	12.02	1.22	<0.001	9.59	14.44
Group (ADHD)	−2.34	0.73	0.002	−3.80	−0.88
Gender (female)	−0.87	0.74	0.240	−2.34	0.60
Cognitive impulsivity	0.60	0.04	0.094	−0.01	0.13
R2 adjusted: 0.15					
**Execution (ROCF)**	**β**	**Standard Error**	***p*-Value**	**95% Confidence Interval**	
				**Lower limit**	**Upper limit**
Intercept	23.78	3.12	<0.001	17.54	30.01
Group (ADHD)	−5.44	1.83	0.004	−9.10	−1.77
Gender (female)	1.07	1.83	0.561	−2.59	4.73
Cognitive impulsivity	0.03	0.09	0.720	−0.14	0.21
R2 adjusted: 0.11					

ZM: Zoo Map A—Behavioral Assessment Disexecutive Syndrome; ROCF: Rey–Osterrieth Complex Figure Test; HM: Hand Movements—K-ABC.
